# Ubiquitous digital technologies and spatial structure; an update

**DOI:** 10.1371/journal.pone.0248982

**Published:** 2021-04-15

**Authors:** Emmanouil Tranos, Yannis M. Ioannides

**Affiliations:** 1 School of Geographical Sciences, University of Bristol, Bristol, United Kingdom; 2 Department of Economics, Tufts University, Medford, MA, United States of America; 3 The Alan Turing Institute, London, United Kingdom; Xiamen University, CHINA

## Abstract

This paper examines the impact of widespread adoption of information and communication technologies (ICT) on urban structure worldwide. Has it offset agglomeration benefits and led to more dispersed spatial structures, or has it strengthened urban externalities and thus resulted in more concentrated spatial structures? Theoretical and empirical studies on this question have produced contradictory findings. The present study recognizes that assumptions made earlier about the evolution of technological capabilities do not necessarily hold today. As cutting-edge digital technologies have matured considerably, a fresh look at this question is called for. The paper addresses this issue by means of several data sets using instrumental variable methods. One is the UN data on Urban Settlements with more than 300, 000 inhabitants. Estimation methods with these data show that increased adoption of ICT has resulted in national urban systems that are less uniform in terms of city sizes and are characterized by higher population concentrations in larger cities, when concentration is proxied the Pareto (Zipf) coefficient for national city size distributions. Two, is disaggregated data for the urban systems of the US, defined as Micropolitan and Metropolitan Areas, and for the UK, defined as Built-up Areas in England and Wales, respectively. These data allow for the impacts to be studied for cities smaller than those included in the cross-country data. Increased internet usage improved a city’s ranking in the US urban system. Similarly, increased download speed improves a built-up area’s ranking in England and Wales.

## Introduction

Geographers, planners and urban economists spent effort in exploring the spatial footprint of the internet even at its early stages. They theorized about the spatial impacts that rapid internet penetration might generate on individual cities and the national spatial structure. The outcome of various such efforts was rather conjectural and even fanciful and not data-based. Cases in point are celebrations of the emergence of telecottages [1], the rise of a borderless world [2], the death of cities [3, 4], and, more generally, the end of geography [5], the death of distance [6] and the emergence of a new flat world [7]. Today, 25 years after the commercialization of the internet [8], we know that the above narratives overstated the potential of the internet and other digital communication technologies to supplement face-to-face interactions, diminish the cost of distance and, indeed weaken agglomeration economies. The high and steadily increasing urbanization rates [9] falsify such predictions.

The adoption rate and the pervasiveness of new internet-based information and communication technologies, ICT for short, such as online social media and mobile-telephony hosted internet, which have increased rapidly during the last 10-15 years in the developing world, too, raise questions about how exactly ICT might have affected agglomeration economies. Conflicting technology examples can be illustrated. On the one hand, despite the broader agreement that no digital technology can reach the content richness of face-to-face communications, empirical research from the management field suggests that current digital technologies can effectively facilitate the sharing of knowledge with low to medium tacitness and even support knowledge sharing of a high degree of tacitness [10]. On the other hand, the very same technologies can further enhance what Storper and Venables [11] termed as buzz, as the constant publication of our personal and professional updates and whereabouts enabled by ICT can directly facilitate deliberate as well as unplanned or even unintentional face-to-face meetings [12].

Urban economics suggests that a key source of agglomeration externalities is proximity, which facilitates interaction and knowledge spillovers [13]. Hence, face-to-face interactions and the implied knowledge spillovers within cities flourish because of lower transportation and interaction costs [11]. However, ICT have the capacity to directly affect this process by further reducing transportation cost [14]. In essence, the internet and digital communications promote further learning and matching, which are at the heart of the micro-foundations of agglomeration economies identified by Duranton and Puga [15]. Web-based applications such as Massive Open Online Courses decrease the need for co-location of actors in order to participate in formal learning activities. Furthermore, online social media such as LinkedIn can enhance the probability of matching and indeed the quality of matches even within cities. This is why LinkedIn has been identified as “the largest professional matchmaker site in the world” [[16] p. 207; [17]]. Therefore, it is natural to ask whether the widespread adoption of ICT could offset the benefits of immediate physical proximity and result in more dispersed spatial structures, or further reinforce agglomeration externalities and lead to more concentrated spatial structures.

This paper contributes to the above discussion by presenting empirical research on whether the internet and digital communications have affected spatial structure, as seen via the size distribution of cities. Contrary to most of the previous empirical studies, which are reviewed in the next section, this paper returns first to an empirical setting similar to that of Ioannides et al. [18] in order to re-examine their results by employing updated data on internet use, associated with the enhanced technological maturity of ICT, and using a number of different data sets. In contrast to Tranos and Ioannides [19], which revisits the question by seeking to replicate Ioannides et al. [18], but with the same but augmented data as that earlier study, the present paper probes the significance of different levels of aggregation for the relation between agglomeration externalities and digital communications by employing different scales of spatial aggregation. One is a global multi-country analysis using urban agglomeration data for many countries; a second is a more granular analysis for the US and the UK. For those two countries the paper brings novel data to bear on the question. In addition, the paper also distinguishes the effects of a broader range of ICT on spatial structure, including internet and broadband internet as well as mobile and landline telephony adoption rates.

Interestingly, in contrast to Tranos and Ioannides [19], most of the differences in the results—from the global level analysis to the case studies—support a complementarity argument. Specifically, the paper examines econometrically whether spatial structures have been affected by the adoption rates of the different digital communication technologies described above. The results are robust against potential endogeneity concerns, as one might claim that the take-up of these technologies could have been affected by spatial structures themselves. That is, individuals living in more dispersed spatial systems might have made greater use of such technologies in order to overcome the lower level of agglomeration externalities. Notably, we find some evidence that such effects are stronger in smaller urban areas. Our findings can directly inform the urban policy agenda as they advocate in favor of including digital strategies in policies aiming to enhance agglomeration externalities and to improve the position of a city within its national urban hierarchy.

The impact of ICT adoption on urban spatial structure is of such paramount importance in understanding its impact on modern economies that replicating a study by extending and updating its data is a worthwhile undertaking. By the same token, it behooves us to examine its robustness by means of alternative data sets. Unlike the data used by Ioannides et al [18] and Tranos and Ioannides [19], the data employed by the current study is arguable more consistent. The structure of the paper is as follows: Section 2 provides a brief literature review; Section 3 describes the methods and the data we use; Section 4 presents the results of the multi-country analysis; Section 5 narrows down to the two case studies, the US and UK; and, Section 6 concludes.

## Literature review

Gaspar and Glaeser [20] were the first to model the effect of telecommunication improvements on the intensity of face-to-face interactions and city size. Their results indicate that technological improvements in telecommunications may lead to increased demand for face-to-face interactions, which will then increase the importance of cities. Their theoretical model allows for a complementary relation between agglomeration externalities and advances in telecommunications. However, their results rest on a critical assumption, namely that face-to-face interactions are superior to any technology-mediated interactions. Were it not for this assumption then the opposite prediction would have followed. As indicated above and as the management literature suggests, face-to-face interactions do not necessarily dominate digitally mediated ones. Certain elements of knowledge sharing can also be achieved by via online interactions [10, 21]. Indeed, to a certain extent, this argument is technology dependent. E.g., current teleconferencing capabilities are much more advanced than the ones available in the late 1990s. Hence, it behooves us to return empirically to the impact on agglomeration externalities from improved telecommunication technologies.

Kolko [22] uses internet diffusion data and identifies a clear complementary link between internet usage and city size. Interestingly, he identifies higher internet domain densities in remote cities which indicates a substitution effect of the internet for longer-distance non-electronic communications. His results are consistent for different measures of internet diffusion (internet domain density and internet take-up). Sinai and Waldfogel [23] approach the same question from the consumers’ point of view and find support for complementarity between internet and cities. They study the link between market size and locally targeted online content and find more online local content in larger markets. However, they also obtain evidence for a substitution effect: holding local online content constant, market size has a negative effect on individual connectivity. Forman et al. [24] examines whether commercial internet adoption is higher in cities than in rural areas. While the former would indicate a complementarity between internet adoption and cities, the latter would reflect a substitutability relation, according to which the internet is used as a means to offset costs and lack of opportunities related to peripheriality. Their results indicate that despite internet adoption by firms with more than 100 employees being greater in smaller urban agglomerations, the adoption of more sophisticated internet-based applications is positively related with city size in 2000. Sohn, Kim, and Hewings [25] compare how information technologies are related to urban spatial structure for Chicago and Seoul. Although they find a clear complementary link for Chicago, this is not the case for Seoul, where information technologies contribute to a more dispersed spatial pattern. Focusing on the municipalities in the province of Barcelona, Pons-Novell and Viladecans-Marsal [26] find a complementary link between individual internet take-up and off-line commercial offerings, but their results cannot safely reject a substitution effect. Craig, Hoang, and Kohlhase [27] focus on internet take-up rates across US states during the period 2000–2011. Their analysis provides suggestive evidence of a complementary role that internet connectivity performs on urban living. Ioannides et al. [18] use country-level data on city size distributions to examine the impact of fixed line telephony on urban structure 1980–2000. Using a panel dataset of spatial dispersion measures, they find robust evidence that an increase in the number of telephone lines per capita encourages the spatial dispersion of population in that they lead to a more concentrated distribution of city sizes. In addition, by the end of their study period the internet has come into use, but their results with internet usage is more speculative but do show that it goes in the same direction.

Focusing on rural areas, Partridge et al. [28] find no evidence that rural distance penalties in the US have substantially changed since 1970*s* indicating that technological changes including the internet and digital communications have not affected spatial structure. Also interesting are the findings of a more recent study by Kim and Orazem [29] on the economic effects of broadband internet in rural areas. They identify a positive effect on new firm location decisions, with the effect being higher in rural areas with larger population and in rural areas adjacent to a metropolitan area. These results suggest a complementary relation between the internet and agglomeration economies.

Moving beyond modelling the direct relationship between ICT and spatial structure, Bekkerman and Gilpin [30] focused on the role of locally based information resources using a dataset about the US libraries during the period 2000–2008. Their results suggest that internet access increases the demand and the value of locally accessible information, and such complementarities are higher in larger metropolitan areas. Anenberg and Kung [31] also identified a complementary relation between the internet and consumption variety in cities by focusing on food truck industry in the US.

Thus, clearly, previous research suggests that the declaration of the “death of distance” has proven to be premature [32]. However, the exact impact of digital communications on spatial structure is still an open question. As Leamer and Storper [33] indicate, the internet can affect both centripetal and centrifugal forces. The only cross country study [18] supports a clear substitution effect, whereas most of the above studies have focused either on the US or on some specific cities. Moreover, most of the above studies examined the complementarity/substitutability question over time periods when the internet and other digital communication technologies were still emerging. For instance, internet penetration in the US in 2000, which was the focus for quite a few of the above studies including Ioannides et al. [18], was just above 50 percent, while in 2016 it reached almost 90 percent [34]. At a global scale internet penetration increased from 7 to 46 percent during the same period [35]. In addition, although email and instant messaging technologies were widespread in the developed world in early 2000*s*, network externalities due to mobile internet and online social media were nowhere close to what we are familiar with today. For instance, Facebook users increased from 1 million in 2004 to more than 1.5 billion in 2015 [36]. Hence, it might have been premature for the spatial economic effects of ICT adoption to have been materialized by the time that most of the above studies were conducted.

Most recently, Tranos and Ioannides [19] return to the setting of Ioannides et al. [18], update the data employed and obtain results that confirm their original findings. That is, the diffusion of fixed telephony has caused more dispersed urban structures worldwide, in other words, greater urban decentralization. Similar causal effects are established for mobile telephony, which are novel in relation to Ioannides et al. [18], who lacked such data, and for the internet, which extends their earlier findings. The robustness of their results is confirmed for such alternative measures of dispersion as the Gini coefficient, the Herfindahl index, and the coefficient of variation. This is notable because several years of additional data were used that pertain to an era of rapid expansion of the internet and web-based technologies, more generally.

The present paper does not address the issue that adoption of ICT may have different effects on urban dispersion on a national scale, that is how far are major urban areas, that is, cities, from one another, versus dispersion within major urban areas, that is urban sprawl. Such an inquiry would require detailed data about patterns of how urban sprawl may hamper or promote economic interaction, given advancements in information technology. We think that data from the Covid-19 pandemic would likely throw light at this issue in the future, as it is clear that not all economic activities might continue in an unencumbered manner; see Dingel and Neiman [37].

## Materials and methods

The main aim of this paper is to return to the estimation of the causal impact of ICT adoption on the spatial dispersion of economic activities and consequently population. It adopts an approach that is carried out at several spatial scales and uses different data than those used by Ioannides et al. [18] and Tranos and Ioannides [19].

We start with a multi-country exercise, which includes both developed and developing countries. Section 3.1 discusses the methods and the different data we use and Section 4 reports the results. Because of limitations related with multi-country urban population data (see discussion below), we complement our analysis with two case studies for the US and the UK urban system for which we have access to much more granular data. Section 3.2 discusses the methods and Section 5 reports the results.

### Multi-country analysis

The multi-country identification strategy is a two-step approach [18]. The first step of our methodology is to estimate the Pareto exponent for a broad sample of countries over time. The Pareto exponent is one of the most widely used measures of spatial dispersion with numerous applications in urban economics and economic geography ([see for example [38–44]). This an appropriate measure of dispersion because of the extreme heterogeneity of the city size distribution and the very good fits normally obtained with such estimations. We also replicate our analysis using other measures of dispersion in S1 Appendix. Our results remain consistent. Skipping details pertaining of the suitability of the approach, which may be found in Ioannides et al. [18] and Tranos and Ioannides [19], we proceed with the estimation of the logarithmic form of:

$$\begin{array}{c} ln\left(rank_{i}\right)=ln\left(S_{0}\right)+\zeta ln\left(Size_{i}\right)+e_{i}. \end{array}$$ (1)

This leads to an estimate of *ζ*, known as the Zipf coefficient, but more appropriately the Pareto exponent, a terminology we will adhere to for the remained of the text, even when *ζ* is estimated to be near 1. The term Zipf coefficient should be reserved for the special case of *ζ* = 1. While much of the literature focuses on the Pareto exponent being close to 1, several estimations of city size distributions lead to estimates of *ζ* that are not necessarily equal to 1, or even near it, in which case we refer to *ζ* as the Pareto, or power law, exponent. Given that our aim here is to estimate *ζ* for a number of countries over time as a measure of dispersion, Eq 1 describes the rank of city *i* in country *c* in year *t*:

$$\begin{array}{c} ln\left(rank_{ict}\right)=ln\left(S_{0ct}\right)+\zeta_{ct}ln\left(Size_{ict}\right)+e_{ict}. \end{array}$$ (2)

The estimation of Eq 2 has typically been performed by Ordinary Least Squares (OLS). Gabaix and Ioannides [45] discuss the downward bias of estimates of Eq 2 using OLS on small samples. Gabaix and Ibragimov [46] propose a practical remedy to correct this bias, which we do adopt in this paper: instead of using the *log* of *rank* of a city *i* in a country *c* in year *t*, they propose to use the *log*(*rank* − 0.5), which has indeed been widely adopted. Researchers working in this area must contend with definitional differences as well as differences in availability of different kinds of data sources. Definitions of cities differ across countries, for political, administrative and legal reasons [see [47], Ch. 8, for issues and pitfalls associated with different definitions]. In contrast to the data drawn from Thomas Brinkhoff’s City Population project [48], which were used by Soo [49], Ioannides et al. [18] and Tranos and Ioannides [19], the multi-country analysis in the present paper is based on the annual population data for urban agglomerations with 300, 000 inhabitants or more from the Department of Economic and Social Affairs of the United Nations [9]. Despite some criticism about the consistency of the urban agglomeration definitions across different countries [50], this is the only available source for yearly, multi-country population data for urban agglomerations [51–53]. The results based on these data are reported and discussed in Section 4.

Table 1 presents the estimated *ζ* coefficients for the panel of countries that the second stage of the analysis focuses on using the UN urban agglomerations data. More detailed and interactive visualisations can be found in S2 Appendix. It becomes evident that there is considerable variation in the estimates of *ζ* across the different countries in the sample. Because of the empirically established heavy upper tail of data for cities and urban agglomerations, the *ζ* coefficient constitutes a convenient measure of dispersion. The larger its absolute value, the thinner the upper tail; equivalently, the larger is the coefficient algebraically, the heavier the upper tail. This key observation is basis for the second step of our methodology.

**Table 1:** Pareto exponents.

Countries	2000	2001	2002	2003	2004	2005	2006	2007	2008	2009	2010	2011	2012	2013	2014	2015	2016	2017	2018
AGO	NA	-0.94	-0.94	-0.94	-0.94	-0.94	-0.93	-0.93	-0.92	-0.92	-0.92	-0.91	-0.90	-0.90	-0.89	-0.90	-0.90	-0.91	NA
ARG	-0.97	-0.97	-0.97	-0.97	-0.97	-0.97	-0.97	-0.97	-0.97	-0.97	-0.97	-0.97	-0.97	-0.98	-0.98	-0.98	-0.98	-0.98	NA
AUS	-0.82	-0.82	NA	NA	NA	-0.83	-0.83	-0.82	-0.82	-0.82	-0.82	-0.82	-0.81	-0.81	-0.81	-0.81	-0.81	-0.80	NA
BGD	-0.65	-0.65	-0.66	-0.67	-0.67	-0.68	-0.69	-0.69	-0.70	-0.70	-0.71	-0.72	-0.72	-0.72	-0.73	-0.73	-0.73	-0.73	NA
BLR	-1.26	-1.26	-1.26	NA	NA	NA	-1.24	-1.23	-1.23	-1.22	-1.22	-1.23	-1.23	-1.23	-1.23	-1.23	-1.23	-1.23	-1.23
BRA	-0.93	-0.93	-0.93	-0.93	-0.93	-0.93	-0.93	-0.93	-0.93	-0.93	-0.94	-0.94	-0.94	-0.94	-0.94	-0.94	-0.94	-0.93	-0.93
CAN	-1.06	-1.05	-1.05	-1.05	-1.05	-1.04	-1.04	-1.04	-1.04	-1.03	-1.03	-1.03	-1.03	-1.02	-1.02	-1.02	-1.02	NA	NA
CHL	-0.73	-0.73	-0.73	-0.74	-0.74	-0.75	-0.75	-0.76	-0.76	-0.76	-0.77	-0.77	-0.77	-0.78	-0.78	-0.78	-0.78	-0.79	NA
CHN	-1.15	-1.16	-1.16	-1.17	-1.17	-1.18	-1.18	-1.18	-1.19	-1.19	-1.19	-1.19	-1.19	-1.20	-1.20	-1.20	-1.20	-1.21	NA
CMR	-0.76	-0.76	-0.76	-0.76	-0.76	-0.77	-0.77	-0.77	-0.77	-0.76	-0.76	-0.76	-0.76	-0.76	-0.77	-0.77	-0.77	-0.77	NA
COD	-0.94	-0.94	-0.95	-0.95	-0.96	-0.96	-0.97	-0.97	-0.98	-0.98	-0.99	-0.99	-0.99	-0.99	-0.99	-0.99	-1.00	-1.00	NA
COL	-0.98	-0.98	-0.98	-0.98	-0.98	-0.98	-0.98	-0.98	-0.98	-0.98	-0.98	-0.98	-0.98	-0.98	-0.98	-0.98	-0.98	-0.98	-0.98
DEU	-1.53	-1.53	-1.53	-1.53	-1.53	-1.52	-1.52	-1.52	-1.51	-1.51	-1.51	-1.51	-1.50	-1.50	-1.50	-1.50	-1.50	-1.50	-1.50
DZA	-1.10	-1.11	-1.12	-1.13	-1.14	-1.15	-1.15	-1.16	-1.16	-1.17	-1.18	-1.18	-1.18	-1.19	-1.19	-1.19	-1.19	-1.20	-1.21
EGY	-0.73	-0.73	-0.73	-0.73	-0.73	-0.73	-0.73	-0.73	-0.72	-0.72	-0.72	-0.72	-0.72	-0.72	-0.72	-0.72	-0.72	-0.71	-0.71
ESP	-0.94	-0.94	-0.93	-0.93	-0.93	-0.93	-0.93	-0.92	-0.92	-0.92	-0.92	-0.92	-0.91	-0.91	-0.91	-0.91	-0.91	-0.90	-0.90
FRA	-1.15	-1.15	-1.15	-1.15	-1.14	-1.14	-1.14	-1.14	-1.14	-1.14	-1.14	-1.14	-1.14	-1.14	-1.13	-1.13	-1.13	-1.13	-1.13
GBR	-1.19	-1.19	-1.19	-1.19	-1.19	-1.19	-1.19	-1.19	-1.18	-1.18	-1.18	-1.18	-1.18	-1.17	-1.17	-1.17	-1.17	-1.17	-1.16
IDN	-1.11	-1.11	-1.12	-1.12	-1.12	-1.13	-1.13	-1.13	-1.13	-1.13	-1.13	-1.13	-1.13	-1.13	-1.13	-1.13	-1.12	-1.12	-1.12
IND	-1.05	-1.06	-1.06	-1.06	-1.07	-1.07	-1.08	-1.08	-1.08	-1.09	-1.09	-1.09	-1.09	-1.09	-1.09	-1.09	-1.09	-1.09	NA
IRN	-1.03	-1.05	-1.07	-1.09	-1.11	-1.13	-1.14	-1.15	-1.16	-1.16	-1.17	-1.17	-1.18	-1.18	-1.18	-1.19	-1.19	-1.19	NA
IRQ	NA	-1.06	-1.07	-1.07	-1.08	-1.07	-1.07	-1.07	-1.05	-1.03	-1.06	-1.10	-1.14	-1.17	-1.20	-1.24	-1.23	-1.23	-1.23
ITA	-1.38	-1.39	-1.39	-1.39	-1.40	-1.40	-1.40	-1.41	-1.41	-1.41	-1.42	-1.42	-1.42	-1.43	-1.43	-1.43	-1.44	-1.44	-1.44
JOR	-0.94	-0.97	-1.00	-1.04	-1.07	-1.09	-1.11	-1.12	-1.13	-1.15	-1.16	-1.17	-1.19	-1.20	-1.22	-1.23	-1.25	-1.26	NA
JPN	-0.77	-0.77	-0.76	-0.76	-0.76	-0.76	-0.76	-0.75	-0.75	-0.75	-0.75	-0.75	-0.75	-0.75	-0.75	-0.75	-0.75	-0.75	-0.74
KAZ	-1.93	-1.91	-1.88	-1.85	-1.83	-1.82	-1.81	-1.79	-1.78	-1.76	-1.72	-1.68	-1.63	-1.59	-1.55	-1.51	-1.47	-1.43	-1.39
KEN	-0.75	-0.76	-0.77	-0.78	-0.79	-0.80	-0.80	-0.81	-0.81	-0.82	-0.82	-0.81	-0.81	-0.81	-0.81	-0.81	-0.81	-0.81	NA
KOR	-1.04	-1.05	-1.06	-1.08	-1.09	-1.10	-1.10	-1.11	-1.11	-1.11	-1.12	-1.12	-1.12	-1.12	-1.12	-1.13	-1.13	-1.13	-1.13
MAR	-1.23	-1.23	-1.23	-1.23	-1.23	-1.23	-1.23	-1.23	-1.24	-1.24	-1.24	-1.24	-1.24	-1.24	-1.24	-1.24	-1.24	-1.24	-1.24
MEX	-1.17	-1.18	-1.18	-1.18	-1.18	-1.18	-1.18	-1.18	-1.18	-1.18	-1.19	-1.19	-1.19	-1.19	-1.19	-1.19	-1.19	-1.19	-1.19
MOZ	-1.15	-1.16	-1.17	-1.18	-1.18	-1.19	-1.20	-1.21	-1.23	-1.24	-1.26	-1.26	-1.27	-1.28	-1.31	-1.34	-1.36	-1.38	NA
MYS	-1.04	-1.03	-1.03	-1.03	-1.03	-1.02	-1.02	-1.02	-1.01	-1.01	-1.01	-1.00	-1.00	-1.00	-0.99	-0.99	-0.99	-0.98	-0.98
NGA	-1.13	-1.16	-1.17	-1.18	-1.19	-1.20	-1.21	-1.22	-1.22	-1.23	-1.23	-1.25	-1.25	-1.26	-1.26	-1.27	-1.27	-1.27	NA
PAK	NA	-0.90	-0.90	-0.90	-0.90	-0.90	-0.90	-0.90	-0.90	-0.90	-0.90	-0.90	-0.89	-0.89	-0.89	-0.89	-0.89	-0.89	NA
PER	-0.85	-0.85	-0.85	-0.85	-0.85	-0.85	-0.85	-0.85	-0.85	-0.85	-0.85	-0.85	-0.85	-0.85	-0.85	-0.85	-0.85	-0.84	-0.84
PHL	-1.13	-1.15	-1.16	-1.17	-1.18	-1.18	-1.21	-1.22	-1.22	-1.23	-1.23	-1.24	-1.24	-1.25	-1.25	-1.25	-1.26	-1.26	NA
POL	-1.85	-1.85	-1.85	-1.85	-1.84	-1.84	-1.83	-1.83	-1.83	-1.82	-1.82	-1.82	-1.81	-1.80	-1.79	-1.78	-1.78	-1.77	-1.76
RUS	-1.46	-1.46	-1.46	-1.46	-1.47	-1.47	-1.47	-1.47	-1.47	-1.47	-1.46	-1.47	-1.47	-1.47	-1.47	-1.47	-1.46	-1.46	-1.46
SAU	-0.97	-0.97	-0.98	-0.98	-0.99	-0.99	-0.99	-0.99	-1.00	-1.00	-1.00	-1.00	-1.00	-1.01	-1.01	-1.01	-1.01	-1.01	-1.01
THA	-1.14	-1.16	-1.18	-1.20	-1.21	-1.23	-1.24	-1.26	-1.27	-1.29	-1.30	-1.30	-1.31	-1.31	-1.32	-1.32	-1.33	-1.33	-1.33
TUR	-1.07	-1.07	-1.06	-1.06	-1.06	-1.06	-1.06	-1.05	-1.05	-1.05	-1.04	-1.04	-1.04	-1.04	-1.04	-1.04	-1.05	-1.05	-1.05
TZA	-0.86	-0.86	-0.86	-0.86	-0.87	-0.87	-0.87	-0.87	-0.88	-0.88	-0.88	-0.88	-0.88	-0.88	-0.88	-0.87	-0.87	-0.87	NA
UKR	-1.53	-1.53	-1.53	-1.53	-1.52	-1.52	-1.52	-1.52	-1.52	-1.52	-1.51	-1.51	-1.51	-1.51	-1.51	-1.51	-1.51	-1.51	-1.51
USA	-0.97	-0.98	-0.99	-1.00	-1.01	-1.01	-1.02	-1.03	-1.03	-1.04	-1.05	-1.05	-1.06	-1.06	-1.07	-1.07	-1.08	-1.08	NA
VNM	-0.85	-0.85	-0.85	-0.85	-0.85	-0.85	-0.85	-0.85	-0.85	-0.85	-0.84	-0.84	-0.84	-0.84	-0.83	-0.83	-0.83	-0.82	-0.82
ZAF	-0.80	-0.81	-0.82	-0.83	-0.84	-0.85	-0.86	-0.87	-0.88	-0.88	-0.89	-0.90	-0.91	-0.92	-0.92	-0.93	-0.94	-0.94	NA

*Note*:

Corrected as per Gabaix and Ibragimov (2011)

The second step of our methodology involves estimating the following empirical model [18]:

$$\begin{array}{c} \zeta_{ct}=\theta_{c}+\delta_{t}+X_{ct}+e_{ct}. \end{array}$$ (3)

The model enable us to estimate the effects of a vector *X* of explanatory variables which are included to account for the spatial structure of country *c* in year *t*. The main variables of interest here are internet and digital communications variables that include: internet users per 100 inhabitants, broadband users per 100 inhabitants, mobile phone users per 100 inhabitants, and fixed phone users per 100 inhabitants. To address a potential omitted variable bias Eq 3 includes country (*θ*_*c*_) and year (*δ*_*t*_) fixed effects; *e*_*ct*_ is the error term. In addition, vector *X* includes several control variables, whose descriptive statistics together with those for the other variables used to estimate 3 are reported in Table 2.

**Table 2:** Descriptive statistics for the multi-country model.

Statistic	N	Min	Mean	St. Dev.	Max
Pareto exponent	950	−1.9	−1.1	0.2	−0.7
Pareto exp. inv. sq. error^a^	950	0.01	0.1	0.05	0.2
Population density	931	2.5	126.2	182.1	1,239.6
Government expenditure (% GDP)	913	1.0	14.7	4.9	30.0
Trade (% GDP)	914	19.1	65.3	33.9	220.4
GDP growth	934	−33.1	4.0	4.2	54.2
GDP per capita (log)	892	630.7	18,049.2	15,432.9	61,391.4
Female labour force (%)	950	7.9	37.8	11.9	55.2
Population	950	5,122,493	116,073,037.0	244,323,372.0	1,392,730,000
Non agriculture value added (% GDP)	930	58.8	90.3	8.2	99.4
Internet users per 100 hab.	916	0.01	32.5	28.2	96.0
Broadband users per 100 hab.	824	0.0	8.8	11.2	44.8
Mobile phone users per 100 hab.	949	0.0	72.9	46.4	191.0
Fixed phone users per 100 hab.	946	0.0	19.8	18.6	68.4

^a^This is the inverse squared standard error of the estimated Pareto exponent, which is used for weighting the observations for the estimation of Eq 3. See Section 4

Referring to the control variables, total country population is an important measure of size, GDP per capita, and GDP growth are intimately related to urbanization and so are population density, and non-agricultural value-added as a share of GDP. Trade, that is conventionally measured as exports plus imports as a share of GDP, is an important time varying measure of trade openness. Government expenditure as a share of GDP may be a proxy of public investment in some countries and government waste in others.

As Table 3 indicates, although there some rather strong correlations among these variables, mobile phone penetration appears to have a distinct character from its fixed phone counterpart: their correlation coefficient is only 0.445. This probably highlights the different composition of the population or infrastructure development patterns in the developing world, where mobile telephony helped overcome the lack of fixed line infrastructure and mobile phone networks are also used as the main way to use the internet [54, 55].

**Table 3:** Correlations between ICT variables.

	Internet	Broadband	Mobile	Fixed
Internet users per 100 hab.	1.00	0.87	0.70	0.66
Broadband users per 100 hab.	0.87	1.00	0.53	0.71
Mobile phone users per 100 hab.	0.70	0.53	1.00	0.28
Fixed phone users per 100 hab.	0.66	0.71	0.28	1.00

The availability of a panel dataset for city sizes across countries enables us to use country fixed effects, which can address potential endogeneity issues related to unobserved country specific characteristics of city size distributions. However, such a strategy does not address potential simultaneity issues. Simply put, internet penetration might be affected by spatial structure, as reflected in Pareto exponents, or both internet penetration and spatial structure might be jointly determined by a third variable. E.g., if a country already has a dispersed spatial structure, internet is particularly suitable in facilitating communication. Potential endogeneity in our specification will prevent us from being able to determine the causal impact of internet and digital communication technologies usage on spatial structure, which is the main aim of this paper. In order to address this problem, we will adopt an instrumented variable strategy. Table 2 also includes the descriptive statistics for the instrumental variable we are using and (*female labour force*), will be discussed in Section 4.

### Case study approach

The above global level analysis is followed by two case studies. They allow us to examine the potential effects of the internet on two mature urban systems in greater detail and depth and without the exogenously imposed threshold of the 300, 000 habitants that the global level analysis affords us. We focus on the US and the UK, for which we are able to utilize more granular internet-related data (see Section 5 for the data description). Given that we are dealing with specific countries and not with a panel of countries we cannot employ an identification strategy similar to that of the global level analysis discussed in the previous section. Therefore, we propose a one-step approach involving estimation of the following empirical cross-sectional model:

$$\begin{array}{c} Diff\;in\;ranks_{i}=a+\beta ICT_{i}+BC_{i}+e_{i}. \end{array}$$ (4)

In order to capture the micro-dynamics of the urban systems in the two case studies, we follow Batty [56] and Havlin [57] and focus on the difference in ranks for individual cities during the study period. Changes in ranking of individuals have also been used elsewhere in economics because of its robustness properties. C.f. Chetty and Hendren [58]. Contrary to their approach, we are interested in the real difference in rankings rather than in absolute differences, in order to capture whether or a city improves its ranking within the urban system during the study period. We then test whether our internet-related variable has an effect on the change in ranking. Hence, we define the left-hand side (LHS) variable of Eq 4 as follows:

$$\begin{array}{c} Diff\;in\;ranks_{i}=r_{i(t-1)}-r_{it}, \end{array}$$ (5)

where *r*_*it*_ is the population rank of city *i* in year *t*. A negative (positive) value for the *Diff in ranks*_*i*_ variable indicates that city’s *i* position in the urban hierarchy of the country worsens (improves) in relative terms, also due to the population changes of the other cities of the urban system. Notably, this variable does not only consider the population change of a specific city, but it also considers the overall urban system dynamics by focusing on the rank and not on population per se. Given that the data we use and the definitions of cities vary between the UK and the US we are going to discuss these data in the relevant sections. What we highlight here is that the estimation of Eq 4, just like Eq 3, might suffer from endogeneity and therefore an instrumental variables strategy is employed in order to address this issue.

## Digital technologies and spatial structure: A global view

This section presents the estimation results of 3. The LHS variable is *ζ*, the Pareto exponent, as estimated according to the Gabaix and Ibragimov [46] correction. The main explanatory variables of interest are provided at the top of Table 4; namely internet, broadband, mobile and fixed telephony per 100 habitants, expressed in natural logarithms. The main variables of interest are introduced successively on their own in the regressions reported in Table 4. All regressions include country fixed effects to control for unobserved heterogeneity and a time trend. In addition, the observations are weighted with the inverse squared standard error of the estimated Pareto exponent (see Table 2) to address potential noise that is carried over from the first part of our identification strategy. In regards to the interpretation of the estimated coefficients, given that the Pareto exponent has entered the regression not as an absolute value, but instead as a real number a positive coefficient for a RHS variable indicates an impact towards the decrease of the spatial dispersion of population. In other words, a positive coefficient indicates an effect towards less uniform city sizes that is more dispersion of city sizes. The latter is indicative of enhancement of agglomeration economies because of the expansion of digital technologies.

**Table 4:** OLS estimation of Eq (3).

	*Dependent variable*:			
	Pareto exponent 2000-18			
	(1)	(2)	(3)	(4)
Internet users per 100 hab. (log)	0.001***			
	(0.0001)			
Broadband users per 100 hab. (log)		−0.001**		
		(0.0002)		
Mobile phone users per 100 hab. (log)			0.0003	
			(0.0003)	
Fixed phone users per 100 hab. (log)				−0.00003
				(0.0001)
Population density (log)	−1.378	−1.060	−1.505	−1.892
	(1.870)	(1.985)	(1.896)	(2.007)
Government expenditure (	(0.001)	(0.001)	(0.001)	(0.001)
Trade (% of GDP)	0.0002***	−0.0001	0.0001	0.0001
	(0.0001)	(0.0001)	(0.0001)	(0.0001)
Non agriculture value added (% GDP)	0.0001	0.001	−0.0001	−0.0002
	(0.001)	(0.001)	(0.001)	(0.001)
GDP growth	0.001***	0.001***	0.001**	0.001**
	(0.0003)	(0.0004)	(0.0004)	(0.0004)
GDP per capita (log)	−0.020***	−0.023***	−0.019***	−0.016**
	(0.007)	(0.006)	(0.007)	(0.008)
Population (log)	1.300	0.955	1.390	1.785
	(1.873)	(1.985)	(1.899)	(2.011)
Constant	−18.795	−13.969	−19.948	−25.516
	(26.314)	(27.886)	(26.670)	(28.261)
Country fixed effects	Yes	Yes	Yes	Yes
Yearly fixed effects	Yes	Yes	Yes	Yes
Observations	844	757	865	867
Adjusted R^2^	0.987	0.988	0.986	0.986
Residual Std. Error	0.516	0.497	0.540	0.540

*Note*:

*p<0.1;

**p<0.05;

***p<0.01

Robust std. errors in parenthesis

Tables 4 and 5 report the estimation results based on the UN urban agglomeration data. Starting with the former, we note that an agglomerative effect is only detected for the internet users and while a marginally opposing result can be seen for broadband users as indicated by the significant coefficients in columns (1) and (2). For the telephony variables the estimation of 3 did not yield statistically significant coefficients. Before discussing further these results and the effects of the other control variables, we need to highlight that the main challenge of estimating Eq 3 is the potential endogenous nature of the share of internet users which might prevent us from being able to infer a truly causal effect. Endogeneity might be an issue here as spatial structure, which is proxied by the Pareto exponent, might be affected by another source, which also affects internet penetration. For instance, economic development might affect the concentration of population in large cities and at the same time enable more people to go online. If we do not address this issue, the coefficient for the main variable of interest will capture potential effects that internet penetration has on spatial structure, but also potential reverse causality effects that spatial structure might generate on internet penetration. To overcome this potential problem, Table 5 reports estimates of Eq 3 using two-stage least squares (2SLS) with instrumental variables (IVs). The latter are variables which are correlated with our endogenous variables, but do not influence current spatial structure. Such an approach will enable us to estimate the causal effect—if any—of the internet and digital communications on spatial structure. At a first stage, our endogenous variables are regressed against the IVs. Then, the predicted values of the endogenous variables based on the IVs and the other control variables are used instead of the endogenous variable to estimate Eq 3. A significant effect will verify the causal impact of the internet and digital communication usage on spatial structure.

**Table 5:** 2SLS estimation of Eq (3).

	*Dependent variable*:			
	Pareto exponent 2000-18			
	(1)	(2)	(3)	(4)
Internet users per 100 hab. (log)	0.001***			
	(0.0004)			
Broadband users per 100 hab. (log)		0.002**		
		(0.001)		
Mobile phone users per 100 hab. (log)			−0.007*	
			(0.004)	
Fixed phone users per 100 hab. (log)				−0.001***
				(0.0003)
Population density (log)	−0.827	−1.736	−12.765	−0.828
	(1.841)	(2.622)	(8.326)	(2.262)
Government expenditure (% GDP)	−0.0003	0.0002	−0.002	−0.0003
	(0.001)	(0.002)	(0.002)	(0.001)
Trade (% of GDP)	0.0004***	0.0004*	0.001	−0.0003
	(0.0001)	(0.0002)	(0.0005)	(0.0002)
Non agriculture value added (% GDP)	0.0003	−0.0003	−0.001	−0.001
	(0.001)	(0.001)	(0.001)	(0.001)
GDP growth	0.001***	0.002***	0.001*	0.0001
	(0.0004)	(0.001)	(0.001)	(0.0004)
GDP per capita (log)	−0.023***	0.003	0.034	−0.0003
	(0.007)	(0.012)	(0.038)	(0.011)
Population (log)	0.776	1.867	12.794	0.740
	(1.841)	(2.646)	(8.410)	(2.267)
Constant	−11.513	−27.468	−180.823	−10.915
	(25.852)	(37.246)	(118.577)	(31.852)
Weak instruments	44.77	22.23	3.51	27.24
Wu-Hausman	3	15.96	12.26	12
P-value	0.08	0	0	0
Country fixed effects	Yes	Yes	Yes	Yes
Yearly fixed effects	Yes	Yes	Yes	Yes
Observations	844	757	865	867
Adjusted R^2^	0.986	0.984	0.944	0.979
Residual Std. Error	0.534	0.579	1.078	0.657

*Note*:

*p<0.1;

**p<0.05;

***p<0.01

Robust std. errors in parenthesis

IV: Female participation in labour force

First stage regressions can be found in S3 Appendix

The main challenge for such an exercise is to find a valid IV. In our case, the challenge is even bigger as we need to find a variable which is not only fit for purpose, but it also contain as few missing values as possible in order to retain the same number of observations as the OLS models. This is particularly difficult for a diverse sample of countries as the one we are dealing with here. We propose here the percentage of female participation in the labor force, which is directly related to digital infrastructure, but not to spatial structure (see Table 2 for the descriptive statistics). The literature provides empirical and theoretical evidence that ICT are correlated with female participation in labour markets mostly because they enable teleworking [59–63] At the same time, female labor participation is not related with spatial structure especially given the diverse sample of countries. Although one might think that increased female labor participation may lead to relocation of households to large cities and therefore affect the spatial structure of cities, such a process varies a lot within our dataset. Even if this might be true for developed countries with mature urban systems for which a location within a large urban center might provide opportunities for both male and female workers to find jobs, our dataset also includes countries, 41 per cent of the GDP of which is attributed to primary activities (see Table 2). So, female labor participation might also be related to economic activities located outside large urban centers. In terms of the relevant tests presented in Table 5 the weak identification first stage F-test exceeds by far all the Stock-Yogo critical values for all, but the mobile telephony regression. Hence, we are confident to interpret the effects of internet, broadband internet and fixed telephony usage.

The estimations presented in Table 5 indicate that increases in internet and broadband internet usage have resulted to a decrease of the spatial dispersion of population for the time period and for the panel of countries included in our data. In other words, increases in internet and digital communications resulted to national urban systems which are less uniform in terms of city sizes and are characterized by higher population concentrations in larger cities. The exact opposite appears to happen for fixed telephony, which our results indicate that has led to an increase of spatial dispersion of population. We expect that the IV estimates capture the local average treatment effect [64] of these labour markets, which are characterised by more active engagement with the internet, and, therefore, experience a larger effect on the Pareto exponents. Importantly, our identification strategy enables us to address potential reverse causality issues and treat the results of Table 5 as causal. Hence, the main finding of our multi-country, global analysis based on data for urban agglomerations of at least 300,000 inhabitants is that internet and broadband internet usage appear to act in favor of agglomeration economies and result to urban systems with more dominant cities on the top of the urban hierarchy. On the contrary, fixed telephony usage acted in favour of centrifugal forces. Our identification strategy does not enable us to make similar interpretations for mobile telephony usage.

Regarding the control variables, only a few of them have significant effects on spatial structure probably because the fixed effects estimation masks the between-country variation. The effects of these control variables are in agreement with previous research (Ioannides et al. 2008). Namely, GDP per capita has a negative effect which indicates that wealthier countries tend to have more balanced urban systems, while the opposite seems to happen for countries experiencing a growing economy.

In total, the results indicate that our measures of internet and telephony penetration have further enhanced agglomeration forces, at least for large urban agglomerations, while the opposite happens for fixed telephony. These results are robust against endogeneity issues, but are limited to urban agglomeration included in our data. Indeed, for the estimation of the Pareto exponent we only included urban agglomeration of 300, 000 inhabitants or more due to data availability. Hence, the above estimations cannot verify whether such an effect is also valid for smaller cities. Interestingly, the above results regarding the role of fixed telephony are in agreement with previous results from [18, 19] which used a smaller panel of countries but without constraints regarding the city size. However, our results do not support the findings of [19] regarding internet usage.

It is indeed puzzling that the results on Table 5 that the effects on urban concentration for internet users and broadband users, on the one hand, and mobile and fixed telephony users are in opposite directions. We wish to broach this subject in the context of some related literature, namely [65, 66]. The latter supports the notion that ICT favors agglomeration in the sense that in spite of their convenience modern technologies do no make up for the need of proximity in social interactions. The former supports the notion people who communicate more frequently are likely to be near one another. ICT, as viewed through such granular studies of individuals’ communications do not make up for physical distance; individuals nearer one another communicate more.

In order to overcome the city size limitation, the next section presents two case studies, for which we have obtained much more granular data and therefore we are in a position to test the effect of the internet on the tail of the urban population distribution for these countries.

## The impact of ICT on the US and the UK spatial structures

This section focuses on the US and the UK urban systems, for the cities of which Eq 4 from Section 3.2 is estimated separately.

### Internet and the US spatial structure: Evidence from the US micropolitan and metropolitan statistical areas, 2013-2018

We pursue further our investigation of the impact of internet adoption by using a previously unutilized, to the best of our knowledge, for this purpose data source. That is, for the first time in 2013, data on internet use was made available via the American Community Survey and is provided at the metropolitan area (urban areas comprised of one or more adjacent counties or county equivalents that have at least one urban core area of at least 50, 000 population, plus adjacent territory that has a high degree of social and economic integration with the core as measured by commuting ties) and at the micropolitan area (defined like metropolitan areas except that are comprised of an urban core of at least 10, 000, but less than 50, 000 population) level of aggregation [67]. These functional definitions of a city represent in essence labor markets and according to Table 6 the observable minimum size of city population used for this analysis is just above 62, 000 habitants.

**Table 6:** Descriptive statistics for the USA model.

Statistic	N	Min	Mean	St. Dev.	Max
difference in ranks, 2013-18	461	−21	0.028	8.171	47
households w. internet 2013, %	461	0.307	0.716	0.073	0.871
population 2013	461	62,282	568,372.100	1,370,757.000	19,949,502
% of unemployment 2013	461	2.300	8.530	2.591	19.900
% of white population 2013	443	0.173	0.806	0.129	0.963
income 2013	461	15,455	24,336.230	3,802.734	41,498
population density 2013	461	0.028	0.918	1.036	9.288
employment in service 2011, %	461	49.000	78.996	6.277	92.300
commute in minutes 2013	461	15	22.428	3.280	38
pop. above 25 w. Bachelor’s degree 2005, %	461	5.600	14.832	4.727	33.900
commute in minutes, 2005	461	14.000	21.739	3.436	40.700

The LHS variable we use here is the outcome of Eq 5. We use this variable to estimate 4 with OLS. Additionally, we employ a normalized form of the dependent variable, so as that variable is bounded between 0 and 1 and with a mean of 0.5:

$$\begin{array}{c} Normalized\;difference\;in\;ranks_{i}=\frac{difference\;in\;ranks_{i}+N_{cities}}{2xN_{cities}}, \end{array}$$ (6)

This transformation enables us to estimate 4 with a quasibinomial GLM estimator given that the original form of our LHS variable does not vary continuously, but is instead defined as a difference between two count variables, which may also assume negative values. The results of the different estimations remain qualitatively the same regardless of the form of the LHS variable we use. Table 7 reports the results of the estimation of Eq 4 using OLS. The sign and significance of the main variable of interest—percentage of population with computer and broadband connection—verifies our global level results. That is, an increase in internet usage improves the position of a city in the US urban system. The results are consistent among the different estimators that is OLS and GLM. In addition, Table 7 also reports the estimates of interaction effects between the share of population with broadband connection with population and population density. Although the interaction terms are not significant, the main effect remains qualitatively unchanged.

**Table 7:** OLS and GLM estimations of Eq (4) for USA.

	*Dependent variable*:			
	Difference in ranks, 2013-18			
	(1)	(2)	(3)	(4)
% of households w. internet 2013	51.540***	51.212***	114.247*	0.201***
	(9.226)	(10.686)	(65.765)	(0.036)
population 2013 (log)	−0.232	−0.223	3.586	−0.001
	(0.407)	(0.429)	(3.764)	(0.002)
% of unemployment 2013	−0.360*	−0.359*	−0.367*	−0.001*
	(0.196)	(0.197)	(0.197)	(0.001)
% of white population 2013	0.085	0.137	−0.237	0.0003
	(3.409)	(3.445)	(3.412)	(0.013)
income 2013 (log)	−11.663***	−11.695***	−11.210***	−0.045***
	(3.812)	(3.829)	(3.847)	(0.015)
population density 2013	−1.056***	−1.464	−0.987***	−0.004***
	(0.328)	(4.568)	(0.328)	(0.001)
% of employment in service 2011	−0.016	−0.016	−0.015	−0.0001
	(0.065)	(0.065)	(0.066)	(0.0003)
commute in minutes 2013	0.261	0.259	0.286	0.001
	(0.174)	(0.175)	(0.177)	(0.001)
% of households w. internet, 2013 x pop. density, 2013		0.528		
		(5.851)		
% of households w. internet, 2013 x population, 2013			−5.236	
			(5.161)	
Constant	83.035**	83.513**	32.486	0.323**
	(36.479)	(36.997)	(64.658)	(0.142)
Observations	443	443	443	443
Adjusted R^2^	0.122	0.120	0.122	
Residual Std. Error	7.561	7.569	7.562	

*Note*:

*p<0.1;

**p<0.05;

***p<0.01

(1)-(3) is based on OLS, (4) on GLM

For the GLM the Normalized diff. in ranks is used

Robust std. errors in parenthesis

Although not directly comparable, the above estimations are in accordance with our global model. Moreover, the potential presence of endogeneity might be a problem here as well. Therefore, Table 8 reports 2SLS estimations. In addition to the strategy employed for the cross-country analysis—that is the inclusion of one instrument for which we have strong reasons to believe is uncorrelated with the error term—data availability allows us to employ a second IV in order to estimate the Sargan over-identification restrictions test (Column 2). The main instrument we propose here is Bachelor’s degrees per inhabitant in 2005. We need to highlight here that due to differences in scale, time and the endogenous variables, different IVs have been utilized for the different sections of the paper. Even if the quality of human capital affect the population growth of a city 10 years later, our LHS variable adopts a systemic understanding of the US urban system as it measures the relative position of a city within the overall urban system instead of its population growth. In other words, a city might experience population growth between two periods, but if other cities have also experienced population growth, this might not affect its relative position within the urban system. Hence, we do not expect that this IV affects the LHS variable. Moreover, when we add the second IV, which in this case is the commute time in minutes also in 2005, we fail to reject the null hypothesis of the Sargan test, something which also adds the validity of our strategy. Furthermore, our results do not suffer from weak identification according to the relevant tests in Table 8.

**Table 8:** 2SLS estimation of Eq (4) for USA.

	*Dependent variable*:	
	Difference in ranks, 2013-18	
	(1)	(2)
% of households w. internet 2013	93.908 ***	86.712 ***
	(21.028)	(21.085)
population 2013 (log)	-0.624	-0.558
	(0.489)	(0.489)
% of unemployment 2013	-0.429 **	-0.417 **
	(0.206)	(0.202)
% of white population 2013	-5.973	-4.944
	(4.748)	(4.629)
income 2013 (log)	-24.364 ***	-22.207 ***
	(6.541)	(6.437)
population density 2013	-1.110 ***	-1.101 ***
	(0.379)	(0.368)
% of employment in service 2011	-0.117	-0.100
	(0.084)	(0.082)
commute in minutes 2013	0.367 *	0.349 *
	(0.192)	(0.188)
Constant	196.828 ***	177.500 ***
	(60.591)	(59.487)
Weak instruments	114.85	62.05
Wu-Hausman	5.23	3.58
P-value	0.02	0.06
Sargan		5.65
P-value		0.02
Observations	443	443
Adjusted R^2^	0.061	0.080
Residual Std. Error	7.819	7.740

*Note*:

*p<0.1;

**p<0.05;

***p<0.01

Robust Std. Errors in parenthesis

IVs: (1) Bachelors degree per hab. in 2005

IVs: (2) Bachelors degree per hab. in 2005, Commute, minutes, 2005

First stage regressions can be found in S3 Appendix

In total, the estimates of Table 8 enable us to identify a causal effect of the share of households with internet connection on the position of a city in the urban system. Specifically, an increase in internet penetration led to an improvement of the position of a city in the US urban hierarchy for the cities and the time period included in our analysis. Everything else being equal, if a city had experienced an increase of 10 per cent of internet penetration, this increase would have improved its relative position by 9 places. As in the global model reported in Section 4, internet penetration appears to work in favor of agglomeration externalities. In regards to the control variables, we can identify a negative effect of income, which is consistent with the effect of GDP per capita in the global model presented in Table 5. During the study period, unemployment rates and the share of white population negatively affected the ranking of micropolitan and metropolitan areas in the US as reflected in the relevant regressions.

### Internet and the UK spatial structure: Evidence from the built-up areas in England and Wales, 2011-2018

The next step in our analysis is to estimate Eq 4 for cities in England and Wales for which we were able to access internet speed micro-data. More specifically, we obtained individual speed internet tests from broadbandspeedchecker.co.uk. This website enables individuals to directly measure their upload and download internet speed. The results of the tests as well as the geo-location of the users are recorded by the website operator and were provided to us in a fully anonymized manner. More discussion about the nature and the validity of this data can be found in the work of Riddlesden and Singleton [68]. The point nature of these individual level data enables us to aggregate them up to any urban level that we are interested in. Given that all the above analyses focused on functional definitions of cities, we adopt here a morphological definition of a city or a town for the UK, which enables us to test the effect of internet on smaller areas irrespective of being part of a wider urban agglomeration. Therefore we aggregate the internet speed data at the level of Built-up Areas (BUA) for England and Wales. This is a ‘bricks and mortar’ approach which refers to land which is “irreversibly urban in character” including villages, towns or cities. Some key characteristics of these areas include: minimum size of 20 hectares; areas with less than 200 meters between them are linked to a single built-up area; larger built-up areas are separated to smaller sub-divisions of built-up areas [69–71].

**Fig 1:**
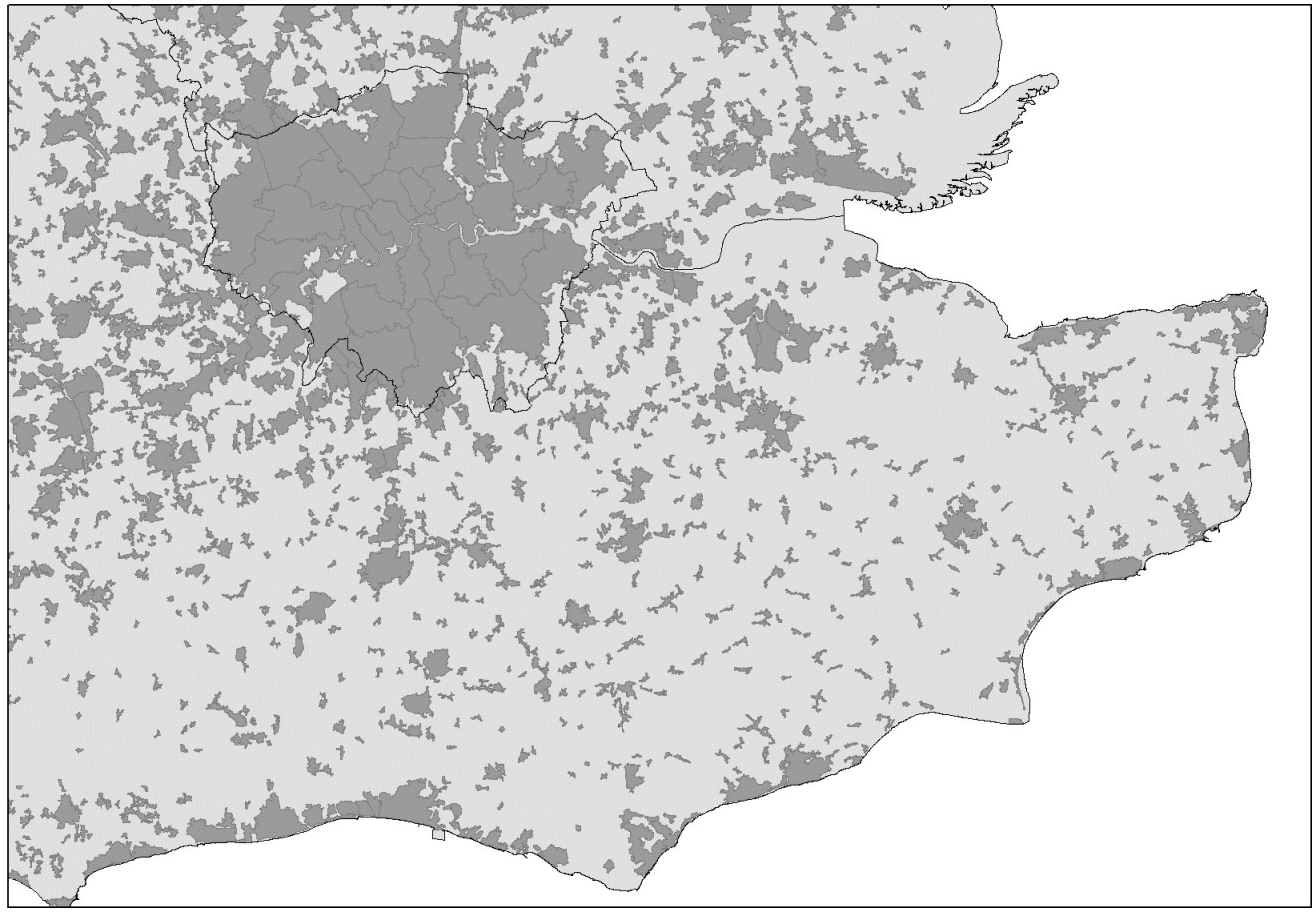
Built-up areas in London and the South East of England.

In order to obtain information about the tail of the urban size distribution we include, wherever available, the sub-divisions of BUA. As Table 9 illustrates, our approach results to 6, 163 observations which include built-up areas and sub-divisions of built-up areas for which we have internet speed data. The lowest population of a built-up area in our data is just above 100 inhabitants in 2011. Fig 1 illustrates the built-up areas we use for the South-East of England and the Greater London Area.

**Table 9:** Descriptive statistics for the UK model.

Statistic	N	Min	Mean	St. Dev.	Max
difference in ranks, 2011-18	6,163	−602	−21.749	70.668	1,235
download speed, 2011	6,163	520	3,745.519	2,523.129	50,280
population, 2011	6,163	101	8,629.648	34,895.950	1,087,558
broadband tests per capita, 2011	6,163	0.0001	0.034	0.062	2.220
% of unemployment, 2011	6,163	0.000	0.048	0.024	0.279
% of British population, 2011	6,163	0.150	0.944	0.063	1.000
population density, 2011	6,163	0.578	24.973	11.607	141.744
employment in service, 2011 (%)	6,163	0.511	0.776	0.054	0.971
Number of universities	6,163	0	0.021	0.245	11

In terms of our identification strategy, we follow the same approach as we did for the US case. Table 10 presents the OLS and GLM estimation of Eq 4 while Table 11 presents the 2SLS estimation. Starting from Table 10 we see a significant and positive effect of the average download speed on the relative position of a BUA in the urban hierarchy in England and Wales. This is consistent with the previous results from the US and also with the global model. Of course, the same endogeneity issues might be present here too and we are addressing that below. In terms of control variables, we include a measure of broadband tests per inhabitant in order to control for potential differences in the take-up of this service. In addition, we include a number of socio-economic variables that we believe can affect the relative position of a city in the urban hierarchy. Unemployment rate has a negative and significant effect as in the US case. The same applies for the percentage of British population, which indicates the importance of migration in urban growth. Population density and the percentage of people working from home also negatively affected the relative position of BUA during 2011−2018. Finally, Table 10 includes two interaction terms (columns 2 and 3) between download speed and population and population density. The negative and significant sign for both interaction coefficients indicates that the effect of download speed decreases as the size of the BUA or its density increases. Contrary to the US case, this is indicative of a larger digital connectivity effect for smaller BUA.

**Table 10:** OLS and GLM estimations of Eq (4) for the UK.

	*Dependent variable*:			
	Difference in ranks, 2013-18			
	(1)	(2)	(3)	(4)
download speed, 2011 (log)	5.484***	14.807***	21.973**	0.0004***
	(2.029)	(5.348)	(8.791)	(0.0002)
population, 2011 (log)	5.720***	5.808***	23.875***	0.0004***
	(1.052)	(1.056)	(8.964)	(0.0001)
broadband tests per capita, 2011	−3.991	−2.247	−2.017	−0.0003
	(10.382)	(10.556)	(10.471)	(0.001)
% of unemployment, 2011	−142.544***	−140.509***	−142.753***	−0.011***
	(53.532)	(53.629)	(53.493)	(0.004)
% of British population, 2011	−34.756**	−42.859**	−39.608**	−0.003**
	(16.009)	(16.687)	(16.466)	(0.001)
population density, 2011	0.008	2.793*	0.011	0.00000
	(0.150)	(1.571)	(0.150)	(0.00001)
% of people working from home, 2011	−80.250**	−71.360*	−74.454*	−0.006**
	(38.392)	(39.203)	(38.381)	(0.003)
employment in service, 2011 (%)	−61.945**	−62.921**	−64.734**	−0.005**
	(28.073)	(28.066)	(28.098)	(0.002)
download speed, 2011 (log) x pop. density, 2011		−0.338*		
		(0.183)		
download speed, 2011 (log) x population, 2011 (log)			−2.175**	
			(1.080)	
Constant	−6.790	−76.679	−138.062*	0.499***
	(34.074)	(48.125)	(73.822)	(0.003)
Observations	3,546	3,546	3,546	3,546
Adjusted R^2^	0.049	0.050	0.049	
Log Likelihood				14,294.670
Akaike Inf. Crit.				−28,571.330
Residual Std. Error	56.940	56.907	56.929	

*Note*:

*p<0.1;

**p<0.05;

***p<0.01

(1)-(3) is based on OLS, (4) on GLM

For the GLM the Normalized diff. in ranks is used

Robust std. errors in parenthesis

**Table 11:** 2SLS estimation of Eq (4) for the UK.

	*Dependent variable*:	
	Difference in ranks, 2011-18	
	(1)	(2)
download speed, 2011 (log)	43.058***	47.226***
	(11.727)	(6.298)
population, 2011 (log)	−1.735	−2.451*
	(2.327)	(1.389)
broadband tests per capita, 2011	−6.658	−5.763
	(12.159)	(12.263)
% of unemployment, 2011	−93.185	−89.366
	(60.397)	(59.332)
% of British population, 2011	−36.963**	−38.269**
	(17.921)	(17.631)
population density, 2011	−0.003	−0.010
	(0.144)	(0.147)
% of people working from home, 2011	−92.525**	−93.787**
	(40.412)	(40.729)
employment in service, 2011 (%)	−47.395*	−48.132*
	(28.089)	(28.448)
Constant	−260.358***	−286.509***
	(81.193)	(51.613)
Weak instruments	6.98	42.03
Wu-Hausman	8.08	40.8
P-value	0	0
Sargan		0.02
P-value		0.9
Observations	3,032	3,032
Adjusted R^2^	−0.069	−0.096
Residual Std. Error	57.001	57.721

*Note*:

*p<0.1;

**p<0.05;

***p<0.01

Robust std. errors in parenthesis

IVs: (1) N.r of universities

IVs: (2) N. of universities, N. of broadband tests, 2011

First stage regressions can be found in S3 Appendix

In regards to IV strategy, we use here the *number of universities* as the main instrument. Universities in England and Wales have been established long time ago and therefore we do not expect them to affect the change in the relative position of built-up areas in 2011. The presence of universities and large student populations tend to be correlated with the quality of internet infrastructure provision [72]. The addition of the second instrument (absolute number of broadband tests in 2011) in column 2 results to a Sargan test, the null hypothesis of which cannot be rejected. Moreover, the estimations reported in Table 11 do not suffer from weak instruments as the relevant test is above the rule of thumb value of 10. The coefficients derived from OLS (Table 10) and 2SLS (Table 11) are always positive and significant, something which advocates in favour of the effect of broadband speed in enhancing agglomeration forces. Interestingly, the magnitude of the 2SLS coefficient (column 1 in Table 11) is almost 8 times higher than the OLS coefficient (column 1 in Table 10). We attribute the OLS underestimation, which is more or less consistent in all our estimations, to the difficulty in directly observing active engagement with ICT. Our endogenous variable captures the quality of the internet infrastructure as the experienced internet speed. Similarly to the endogenous variables of the global and the US model (internet and telephony take-up), what these variables do not capture is what individuals actually do with ICT [73]. For example, if we assume a bi-modal internet usage for either entertainment or work purposes, one would expect that the latter would have a larger effect on spatial structure than the former. We believe that our endogenous variables capture mostly the former. Therefore, the IV estimations, especially for UK case, reveal the local average treatment effect [64] by capturing the effect of these users/households who actively engage with ICT (e.g. work on ICT-related sectors) and, therefore, have a higher effect on spatial structure. A back of the envelope calculation indicates that, everything else being equal, 10 per cent increase in download speed for a BUA, would have increased its position by 4 places.

In total, the results of the UK case study are in accordance with the previous results of the global, multi-country analysis and of the US urban system. Interestingly, the positive effect of internet is still apparent when the analysis adopts a morphological definition of cities and when the emphasis is not on internet penetration, but instead on the quality of internet infrastructure as reflected in download broadband speed. What is also interesting for the UK case study is that this effect appears to be higher for small and less dense BUA.

## Discussion and conclusions

This paper reports empirical findings on a question at the heart of urban economics and economic geography: has the proliferation of information and communication technologies offset the benefits of agglomeration economies and resulted in more dispersed spatial population structures, or has it further reinforced such urban externalities and led to more concentrated spatial structures? Previous studies have led to contradictory results regarding whether ICT adoption and urban agglomeration externalities are complementary or substitutable. As Leamer and Storper [33] stress, the internet can affect both centripetal and centrifugal forces. The present study revisits Ioannides et al. [18] with a completely open mind in view of the availability of several years of additional data on internet penetration across the world. It examines the robustness of the findings of Tranos and Ioannides [19] by employing alternative data sets. In general, quite a few earlier studies have either been based on assumptions about technological capabilities that might no longer hold today, or use data that do not fully capture the widespread adoption and maturity of communication technologies that has taken place since the data coverage of those earlier studies.

We report estimation results using international country-level data on urban agglomerations with more than 300, 000 inhabitants from the UN Urban Settlements data and test the effect of ICT adoption on the Pareto exponents for national city size distributions, as measures of dispersion for heavy-tailed data. We report results which are robust to endogeneity concerns. Then, in order to examine such effects for cities that are smaller than those included in our international data, we focus on the US and the UK urban systems. Specifically, we test the effect of internet usage and internet speed on the changes over time in rankings of Micropolitan and Metropolitan Areas in the US, and of Built Up Areas in the UK. The results favor a complementary relation between the internet and agglomeration externalities. While the cross-country estimates indicate that increase adoption rates of such technologies has resulted in less dispersed urban spatial structures, the two case studies reveal that internet adoption and internet speed improved the relative population rank of a city within its urban system. Interestingly, the latter results indicate that such effects might be even stronger for smaller and less dense urban areas at least in the UK. On the contrary, our estimates regarding the effect of fixed telephony advocate towards the reverse effect: that is fixed telephony acting against of agglomeration forces.

While our results are in agreement with Ioannides et al. [18] regarding fixed telephony, they contradict Tranos and Ioannides [19] The latter cannot of course be ignored. We believe strongly that the differences lie in the different definitions of urban areas. To this date, and in spite of numerous efforts by teams worldwide, there has not been a universally accepted definition, which could moreover be implemented consistently by governments worldwide. The increasingly popular reliance on high-resolution, pioneered by Rozenfeld et al. [74], and satellite-based lights data, most recently used by Duben and Krause [75], for measuring city sizes, are very exciting developments, but they have not yet been accompanied by complementary information on ICT adoption at a comparably granular level. A promising new development is the EU’s Global Human Settlements Initiative and the associated spatial urban data sets; see Pesaresi et al. [76]. Barring the availability of such data, scholars must be careful in drawing conclusions. Arguably, the measurement of the impact of ICT at the level of agglomerations, on one hand, and on changes in rankings, on the other, reported by this paper constitute a contribution to the literature.

We believe that our results, apart from their theoretical value, have the capacity to inform urban policy. The ability of the internet and digital communications to further enhance agglomeration economies can be used as a tool to support urban growth. In addition, the indication that such effects might be stronger for smaller and less dense urban areas, at least in England and Wales, might be helpful to further orient digital strategies towards such locations. Of course, improvements in internet speed is not a trivial policy instrument as it involves numerous complexities. Apart from infrastructure installation costs and engineering challenges, governance issues regarding the ownership of such digital networks as well as the provision of state subsidies create obstacles for the inclusion of such strategies in the urban growth agenda. To further inform such policies, more research at granular scales is needed in order to shed lights on the micro-mechanisms behind such urban processes.

## Supporting information

S1 Appendix (PDF)

S2 Appendix (HTML)

S3 Appendix (PDF)
