# Supplementary material for: Ubiquitous digital technologies and spatial structure; an update
Source: PLoS One. 2021 Apr 15;16(4):e0248982. doi: 10.1371/journal.pone.0248982 (PMC8049296; doi:10.1371/journal.pone.0248982)
Supplement: S1 Appendix — (PDF) [file pone.0248982.s001.pdf]

S1 Appendix

Table 1: 2SLS estimation of equation (3), alt. specifications (i)

	Dependent variable:			
	Gini coefficient 2000-18			
	(1)	(2)	(3)	(4)
Internet users per 100 hab. (log)	0.0003**			
	(0.0001)			
Broadband users per 100 hab. (log)		0.0004		
		(0.0003)		
Mobile phone users per 100 hab. (log)			−0.002	
			(0.001)	
Fixed phone users per 100 hab. (log)				−0.0002**
				(0.0001)
Population density (log)	0.283	0.503	−2.245	0.239
	(0.787)	(0.911)	(2.172)	(0.879)
Government expenditure (% GDP)	−0.0003	−0.0003	−0.001	−0.0003
	(0.0002)	(0.0005)	(0.001)	(0.0003)
Trade (% of GDP)	0.0001**	0.0001	0.0002	−0.00002
	(0.00004)	(0.0001)	(0.0001)	(0.00004)
Non agriculture value added (% GDP)	0.0004**	0.0003	0.0002	0.00004
	(0.0002)	(0.0004)	(0.0003)	(0.0002)
GDP growth	0.0003***	0.001***	0.0003**	0.0001
	(0.0001)	(0.0002)	(0.0001)	(0.0001)
GDP per capita (log)	0.003	0.007*	0.015	0.007**
	(0.002)	(0.004)	(0.010)	(0.003)
Population (log)	−0.298	−0.488	2.249	−0.259
	(0.787)	(0.917)	(2.193)	(0.880)
Constant	4.719	7.291	−31.153	4.190
	(11.046)	(12.896)	(30.910)	(12.363)
Weak instruments	44.77	22.23	3.51	27.24
Wu-Hausman	0.35	4.78	7.09	3.4
P-value	0.55	0.03	0.01	0.07
Country fixed effects	Yes	Yes	Yes	Yes
Yearly fixed effects	Yes	Yes	Yes	Yes
Observations	844	757	865	867
Adjusted R^2	0.991	0.991	0.978	0.990
Residual Std. Error	0.177	0.184	0.279	0.192

Note: *p<0.1; **p<0.05; ***p<0.01
Robust Std. Errors in parenthesis
IV: Female participation in labour force

Table 2: 2SLS estimation of equation (3), alt. specifications (ii)

	Dependent variable:			
	Herfindahl-Hirschman coefficient 2000-18			
	(1)	(2)	(3)	(4)
Internet users per 100 hab. (log)	0.0003^{***}			
	(0.0001)			
Broadband users per 100 hab. (log)		0.0003^{*}		
		(0.0002)		
Mobile phone users per 100 hab. (log)			-0.002^{*}	
			(0.001)	
Fixed phone users per 100 hab. (log)				-0.0002^{***}
				(0.0001)
Population density (log)	0.302	0.691^{***}	−2.240	0.257
	(0.330)	(0.242)	(1.644)	(0.380)
Government expenditure (% GDP)	-0.0004^{**}	-0.0004^{**}	-0.001^{*}	-0.0004^{**}
	(0.0002)	(0.0002)	(0.0004)	(0.0002)
Trade (% of GDP)	0.00003	−0.00002	0.0001	-0.0001^{***}
	(0.00004)	(0.00004)	(0.0001)	(0.00003)
Non agriculture value added (% GDP)	0.001^{***}	0.0004^{*}	0.0004^{*}	0.0003^{*}
	(0.0002)	(0.0002)	(0.0002)	(0.0002)
GDP growth	0.0002^{**}	0.0003^{***}	0.0002	−0.00004
	(0.0001)	(0.0001)	(0.0001)	(0.0001)
GDP per capita (log)	-0.003^{*}	0.001	0.009	0.002
	(0.001)	(0.002)	(0.008)	(0.002)
Population (log)	−0.300	-0.678^{***}	2.258	−0.264
	(0.329)	(0.247)	(1.661)	(0.379)
Constant	4.587	9.865^{***}	−31.462	4.085
	(4.609)	(3.475)	(23.430)	(5.317)
Weak instruments	44.77	22.23	3.51	27.24
Wu-Hausman	12.3	1.57	13.1	9.53
P-value	0	0.21	0	0
Country fixed effects	Yes	Yes	Yes	Yes
Yearly fixed effects	Yes	Yes	Yes	Yes
Observations	844	757	865	867
Adjusted R^2	0.998	0.999	0.995	0.998
Residual Std. Error	0.135	0.102	0.213	0.139

Note: *p<0.1; **p<0.05; ***p<0.01
Robust Std. Errors in parenthesis
IV: Female participation in labour force

Table 3: 2SLS estimation of equation (3), alt. specifications (iii)

	Dependent variable:			
	Coefficient of variation 2000-18			
	(1)	(2)	(3)	(4)
Internet users per 100 hab. (log)	0.005^{***}			
	(0.001)			
Broadband users per 100 hab. (log)		0.011^{***}		
		(0.003)		
Mobile phone users per 100 hab. (log)			-0.030^{*}	
			(0.017)	
Fixed phone users per 100 hab. (log)				-0.004^{***}
				(0.001)
Population density (log)	−2.038	−7.332	−50.695	−2.425
	(3.390)	(6.135)	(31.080)	(4.879)
Government expenditure (% GDP)	−0.003	-0.011^{***}	−0.010	−0.004
	(0.002)	(0.003)	(0.008)	(0.003)
Trade (% of GDP)	0.001^{*}	0.001^{**}	0.002	-0.002^{***}
	(0.0004)	(0.001)	(0.002)	(0.0005)
Non agriculture value added (% GDP)	0.006^{***}	0.001	0.002	−0.0002
	(0.002)	(0.003)	(0.004)	(0.002)
GDP growth	0.003^{***}	0.004^{***}	0.002	-0.002^{*}
	(0.001)	(0.001)	(0.003)	(0.001)
GDP per capita (log)	0.012	0.118^{***}	0.237	0.098^{***}
	(0.017)	(0.029)	(0.157)	(0.025)
Population (log)	2.152	8.075	51.157	2.416
	(3.382)	(6.183)	(31.414)	(4.883)
Constant	−29.411	−114.544	−719.863	−32.802
	(47.450)	(87.025)	(443.067)	(68.597)
Weak instruments	44.77	22.23	3.51	27.24
Wu-Hausman	51.51	50.45	107.28	73.17
P-value	0	0	0	0
Country fixed effects	Yes	Yes	Yes	Yes
Yearly fixed effects	Yes	Yes	Yes	Yes
Observations	844	757	865	867
Adjusted R^2	0.984	0.980	0.838	0.968
Residual Std. Error	1.234	1.402	3.890	1.729

Note: *p<0.1; **p<0.05; ***p<0.01
Robust Std. Errors in parenthesis
IV: Female participation in labour force
