# Supplementary material for: Ubiquitous digital technologies and spatial structure; an update
Source: PLoS One. 2021 Apr 15;16(4):e0248982. doi: 10.1371/journal.pone.0248982 (PMC8049296; doi:10.1371/journal.pone.0248982)
Supplement: S2 Appendix — (HTML) [file pone.0248982.s002.html]

S2 Appendix


# S2 Appendix

# Pareto exponents

The below maps plot the Pareto exponents corrected as per Gabaix and Ibragimov (2011) for all the countries included in our data set for the years 2000, 2005, 2010 and 2010. The quantiles of the yearly exponent distribution are used for the maps with the first quantile always representing the countries with the of the smallest Pareto exponent.

The Pareto exponents are plotted below as an interactive line graph.
