# Supplementary material for: Ubiquitous digital technologies and spatial structure; an update
Source: PLoS One. 2021 Apr 15;16(4):e0248982. doi: 10.1371/journal.pone.0248982 (PMC8049296; doi:10.1371/journal.pone.0248982)
Supplement: S3 Appendix — (PDF) [file pone.0248982.s003.pdf]

S3 Appendix

First stage regressions for the 2SLS estimations

Global model

Table 1: First stage regressions for Table 5

	Dependent variable:			
	Internet users	Broadband users	Mobile phone users	Fixed phone users
	(1)	(2)	(3)	(4)
Female labour force (%)	1.490***	0.696***	−0.280*	−2.040***
	(0.223)	(0.148)	(0.150)	(0.391)
Population density (log)	−598.644	379.944	−1,511.189***	770.219
	(499.831)	(292.430)	(402.110)	(522.031)
Government expenditure (% GDP)	0.283	0.759***	−0.291*	−0.528
	(0.287)	(0.164)	(0.172)	(0.587)
Trade (% of GDP)	−0.202***	−0.141***	0.087***	−0.392***
	(0.038)	(0.021)	(0.030)	(0.073)
Non agriculture value added (% GDP)	−0.450	0.261	−0.045	−0.902**
	(0.280)	(0.200)	(0.141)	(0.381)
GDP growth	−0.442***	−0.269***	0.042	−0.648***
	(0.127)	(0.058)	(0.090)	(0.201)
GDP per capita (log)	10.580***	−4.975***	5.716**	7.812*
	(2.566)	(1.495)	(2.377)	(4.470)
Population (log)	564.464	−456.424	1,528.552***	−759.195
	(500.326)	(292.870)	(404.067)	(524.060)
Constant	−7,950.049	6,596.284	−21,536.980***	10,773.260
	(7,025.000)	(4,113.831)	(5,685.467)	(7,356.142)
Country fixed effects	Yes	Yes	Yes	Yes
Yearly fixed effects	Yes	Yes	Yes	Yes
Observations	844	757	865	867
Adjusted R^2	0.951	0.929	0.951	0.915
Residual Std. Error	189.094	97.068	121.132	371.310

Note: *p<0.1; **p<0.05; ***p<0.01
Robust Std. Errors in parenthesis

Table 2: F tests for IV (female labour force (%)) from first stage regressions for Table 5

	F	p
internet users	75.726	0
broadband users	57.339	0
mobile phone users	7.066	0.008
fixed telephony users	39.818	0

US model

Table 3: First stage regressions for Table 8

	Dependent variable:	
	% of households w. internet 2013	
	(1)	(2)
bachelors degree per hab. 2005	0.007***	0.007***
	(0.001)	(0.001)
commute in minutes 2005		0.003
population 2013 (log)	0.002	0.002
	(0.003)	(0.003)
% of unemployment 2013	0.004***	0.005***
	(0.001)	(0.001)
% of white population 2013	0.158***	0.162***
	(0.029)	(0.029)
income 2013 (log)	0.211***	0.215***
	(0.024)	(0.024)
population density 2013	−0.001	−0.001
	(0.002)	(0.002)
% of employment in service 2011	0.001*	0.001
	(0.0004)	(0.0004)
commute in minutes 2012	−0.0003	−0.003***
	(0.001)	(0.001)
Constant	−1.765***	−1.801***
	(0.240)	(0.240)
Observations	443	443
Adjusted R^2	0.667	0.670
Residual Std. Error	0.041	0.041
Note:		*p<0.1; **p<0.05; ***p<0.01
		Robust Std. Error in parenthesis

Table 4: F tests for IVs from first stage regressions for Table 8

	F	p
bachelors degree per hab., 2005	129.773	0
the above + commute in minutes, 2005	67.772	0

UK model

Table 5: First stage regressions for Table 11

	Dependent variable:	
	download speed, 2011 (log)	
	(1)	(2)
N. of universities	-0.089^{***}	0.029
	(0.033)	(0.018)
N. of broadband tests, 2011		-0.0002^{***}
		(0.00002)
population, 2011 (log)	0.175^{***}	0.233^{***}
	(0.011)	(0.013)
broadband tests per capita, 2011	-0.195	0.083
	(0.186)	(0.147)
% of unemployment, 2011	-0.816	-1.025^{*}
	(0.553)	(0.548)
% of British population, 2011	0.213^{*}	-0.120
	(0.130)	(0.133)
population density, 2011	0.002^{*}	0.002
	(0.001)	(0.001)
% of people working from home, 2011	0.383	0.708^{**}
	(0.299)	(0.298)
employment in service, 2011 (%)	0.203	0.158
	(0.221)	(0.220)
Constant	6.298^{***}	6.191^{***}
	(0.267)	(0.263)
Observations	3,032	3,032
Adjusted R^2	0.208	0.227
Residual Std. Error	0.498	0.492
Note:	*p<0.1; **p<0.05; ***p<0.01	
	Robust Std. Errors	

Table 6: F tests for IVs from first stage regressions for Table 8

	F	p
N. of universities	9.992	0.002
the above + N. of broadband tests, 2011	41.799	0
